# Proteomic identification of the proteins related to cigarette smoke-induced cardiac hypertrophy in spontaneously hypertensive rats

**DOI:** 10.1038/s41598-020-75429-3

**Published:** 2020-11-02

**Authors:** Yuki Kitamura, Nathan Mise, Yurie Mori, Yuka Suzuki, Tomoki Ohashi, Saeko Tada-Oikawa, Masaki Tokisu, Cai Zong, Shinji Oikawa, Sahoko Ichihara

**Affiliations:** 1 Department of Environmental and Molecular Medicine, Mie University Graduate School of Medicine, Tsu, Japan; 2 Department of Environmental and Preventive Medicine, Jichi Medical University School of Medicine, 3311-1 Yakushiji, Shimotsuke, Tochigi 329-0498 Japan; 3 Graduate School of Regional Innovation Studies, Mie University, Tsu, Japan; 4 Department of Occupational and Environmental Health, Tokyo University of Science, Noda, Japan; 5 Present Address: Kinjo Gakuin University School of Pharmacy, Nagoya, Japan; 6 Present Address: Sugiyama Jogakuen University School of Life Studies, Nagoya, Japan

**Keywords:** Proteomics, Cardiology, Risk factors, Biomarkers

## Abstract

Smoking increases the risk of cardiovascular diseases. The present study was designed to determine the effects of 2-month exposure to cigarette smoke (CS) on proteins in the left ventricles of spontaneously hypertensive rats (SHR) and to identify the molecular targets associated with the pathogenesis/progression of CS-induced cardiac hypertrophy. SHR and Wistar Kyoto rats (WKY) were exposed to CS at low (2 puffs/min for 40 min) or high dose (2 puffs/min for 120 min), 5 days a week for 2 months. Using the two-dimensional fluorescence difference gel electrophoresis combined with MALDI-TOF/TOF tandem mass spectrometry, we compared differences in the expression levels of proteins in the whole left ventricles induced by long-term smoking. High-dose CS mainly caused cardiac hypertrophy in SHR, but not WKY, but no change in blood pressure. Proteomic analysis identified 30 protein spots with significant alterations, with 14 up-regulated and 16 down-regulated proteins in the left ventricles of CS-exposed SHR, compared with control SHR. Among these proteins, two members of the heat shock proteins (HSP70 and HSP20) showed significant up-regulation in the left ventricles of CS high-dose SHR, and the results were confirmed by western blot analysis. Our findings suggested that HSPs play an important role in regulation of CS-induced cardiac hypertrophy.

## Introduction

Cardiovascular diseases have remained among the leading causes of morbidity and mortality worldwide^1,2^. Cardiac hypertrophy is a cause of heart failure and an independent predictor of cardiovascular events^3^. Long-term high stress on the heart induces compensatory hypertrophy of the left ventricular (LV) myocardium, increase in cardiac muscle mass, mainly due to increased cardiomyocyte size^4–6^. These pathological processes are mediated through complex series of transcriptional, signaling, structural, electrophysiological and functional events that affect all cardiac cell types^7–9^.

Cardiac hypertrophy is the cellular response to an increase in biomechanical stress, and high blood pressure is one of the best-known risk factor for cardiac hypertrophy^10^. Cigarette smoking is also known as a risk factor for cardiovascular disease^11,12^. It has reported that exposure to cigarette smoke (CS) is associated with increase in blood pressure^13–15^. Synergistic effect between elevated blood pressure and CS exposure has also been reported in epidemiological studies^16^. In addition, CS exposure has been shown to induce cardiovascular oxidative stress^17^.

Spontaneously hypertensive rats (SHR) have been used as a well-established model of genetic hypertension. They are useful to study the transition of hypertension-induced cardiac hypertrophy to heart failure, because they exhibit several features that reflect those in the human heart under conditions of hypertension^18,19^. SHR exhibit progressive LV hypertrophy over the first 9 months of age, followed by depressed myocardial contractile function and ventricular fibrosis by 12 months of age^20,21^. It has been reported that progression of hypertension-induced cardiac hypertrophy was accelerated in SHR exposed to CS^22^.

Proteomics is the technology to identify and quantify overall proteins present content of a cell, tissue or an organism^23^. Proteomics analysis is used to complete complement of proteins expressed by a biological system under different physiological and pathological conditions^24^. In the present study, a proteomics approach was employed to screen for proteins involved in the development of CS exposure-induced cardiac hypertrophy in SHR. Specifically, we determined changes in the expression of proteins in cardiac tissues of hypertrophy in order to find possible molecular mechanisms of cardiac hypertrophy induced by CS.

## Results

### Changes in body and organ weights

The body weight of SHR was significantly lower than that of Wistar Kyoto rats (WKY). WKY and SHR were exposed to CS at low (2 puffs/min for 40 min/day) or high dose (2 puffs/min for 120 min/day) for 5 days a week for 2 months. At the end of the 2-month exposure period, body weight was significantly lower in WKY exposed to CS at high dose (378 ± 7.2 g) than the control (pre-exposure WKY: 351 ± 6.3 g, Table 1). There was no significant difference in body weight between the WKY exposed to CS at low dose and the control. Body weight was significantly lower in SHR exposed to CS at low and high dose than the control group (306 ± 5.4 vs. 278 ± 3.8 or 264 ± 3.0 g, respectively). Furthermore, exposure to CS at high dose increased lung weight and reduced liver weight in SHR only (Table 1).

**Table 1:** Body and organ weights before and after 2-month exposure to low- and high-dose cigarette smoking.

Group	WKY CTL	WKY low	WKY high	SHR CTL	SHR low	SHR high
Body weight, g	378 ± 7.2	363 ± 8.2	351 ± 6.3*	306 ± 5.4*	278 ± 3.8*^,†^	264 ± 3.0*^,†^
Heart weight, mg	11.2 ± 0.14	11.5 ± 0.26	11.9 ± 0.33	12.7 ± 0.32*	12.2 ± 0.17*	13.3 ± 0.33*
Heart weight/body weight, mg/kg	29.7 ± 0.46	31.7 ± 1.08	33.9 ± 1.16*	41.3 ± 0.67*	44.0 ± 0.29*	50.3 ± 1.01*^,†^
LV weight, mg	8.3 ± 0.14	8.4 ± 0.09	8.7 ± 0.14	10.2 ± 0.23*	9.9 ± 0.16*	10.8 ± 0.17*^,†^
LV weight/body weight, mg/kg	22.1 ± 0.36	23.1 ± 0.43	24.9 ± 0.67*	33.2 ± 0.55*	35.6 ± 0.29*^,†^	40.8 ± 0.63*^,†^
Lung weight, mg	12.2 ± 0.25	12.3 ± 0.28	12.8 ± 0.33	10.8 ± 0.27	11.0 ± 0.38	12.8 ± 0.68^†^
Liver weight, mg	114 ± 4.2	112 ± 3.8	107 ± 3.7	113 ± 3.8	104 ± 1.5	96.4 ± 1.2*^,†^
Kidney weight, mg	24.0 ± 0.40	23.5 ± 0.40	23.3 ± 0.44	24.1 ± 0.68	21.9 ± 0.34*	21.0 ± 0.33*

Data are mean ± SEM of seven animals per group.

Low and high represent the dose of inhaled cigarette smoke.

Comparisons among three groups were tested using one-way analysis of variance (ANOVA) followed by Dunnett’s multiple comparison tests using the JMP 8.0 software (SAS Institute Inc, Cary, NC).

*WKY* Wistar Kyoto rats, *SHR *Spontaneously Hypertensive rats, *CTL *control, *LV *left ventricular.

*P < 0.05 versus the WKY CTL group. ^†^*P* < 0.05 versus the SHR CTL group.

### Effect of cigarette smoke on blood pressure and cardiac phenotype

**Figure 1:**
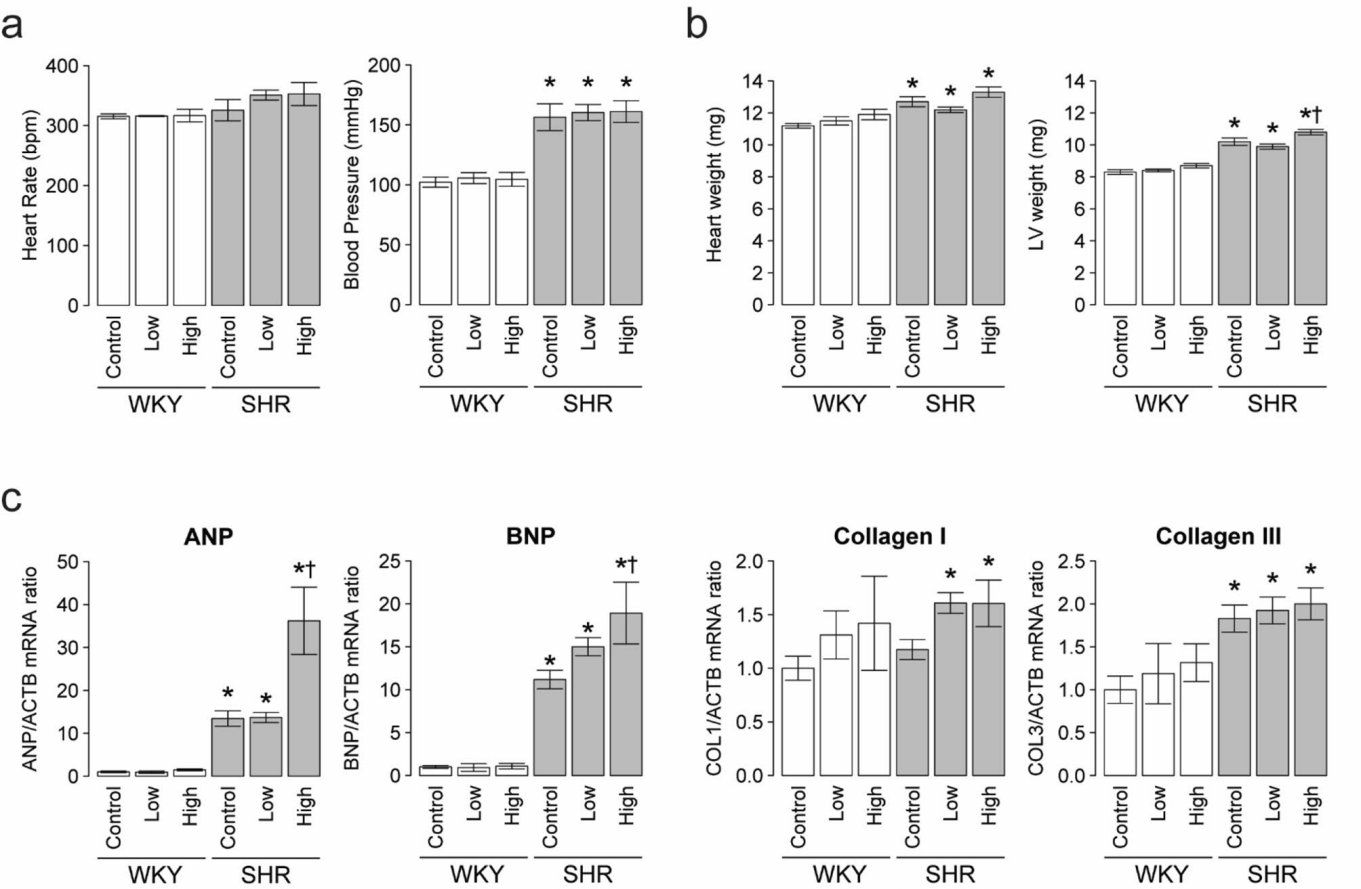
Effects of two doses of cigarette smoke on physiological data and cardiac gene expression. (**a**) Heart rate and systolic blood pressure, (**b**) heart weight and LV weight. (**c**) The mRNA levels of ANP, BNP, collagen I, and collagen III in LV tissues were determined by quantitative RT-PCR analysis. Data are relative to β-actin (ACTB) mRNA expression. Quantitative data are expressed relative to the values for WKY controls. Data are mean ± SEM of 7 rats per group. *P < 0.05 versus the WKY control group. ^†^*P* < 0.05 versus the SHR control (before treatment) group. Comparisons among three groups were tested using one-way analysis of variance (ANOVA) followed by Dunnett’s multiple comparison tests using the JMP (version 8.0) software (SAS Institute Inc, Cary, NC) (https://www.jmp.com/en_us/offers/statistical-analysis-software.html).

There was no significant difference in heart rate after exposure to CS in all groups (Fig. 1a). Systolic blood pressure was significantly higher in control SHR than control WKY. Exposure to CS at the low or high dose did not affect blood pressure in both strains (Fig. 1a). In the control rats, the heart and LV weights were significantly higher in SHR than WKY, and LV weight was significantly heavier in SHR exposed to high-dose CS than the control group (10.2 ± 0.23 vs. 10. 8 ± 0.17 g, Fig. 1b). In both strains, the heart weight/body weight was significantly higher in the CS high-dose rats than control rats (Table 1). The LV weight/body weight was significantly higher in CS high-dose rats than control rats in both strains and the LV weight/body weight was significantly higher in CS low-dose SHR, but not WKY, than control rats (Table 1).

### Effect of cigarette smoke on myocardial hypertrophy and fibrosis

Before exposure to CS, the abundance of ANP and BNP mRNAs in the LV tissues was significantly greater in SHR than WKY (Fig. 1c). ANP and BNP mRNA expression levels in the LV tissues were significantly higher in CS high-dose SHR than SHR control rats, but this was not seen in WKY (Fig. 1c). Collagen I and III mRNA levels in the LV tissues were significantly higher in SHR than in WKY, but there were no significant differences in the levels of these mRNA after exposure to CS in both strains (Fig. 1c).

**Figure 2:**
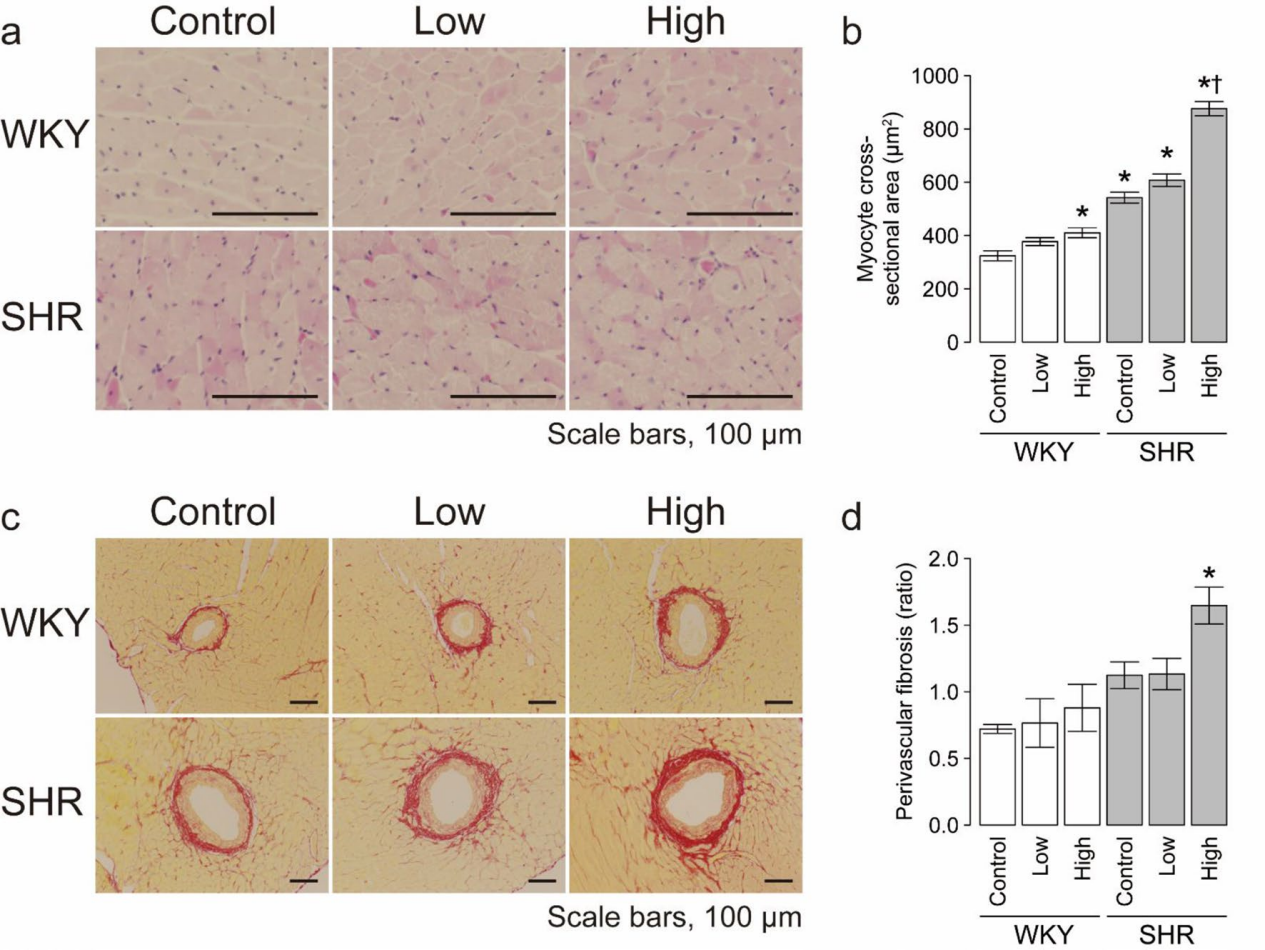
Histological analysis in six groups. Light micrographs of (**a**) myocytes in hematoxylin–eosin-stained sections and (**c**) perivascular fibrosis in Sirius red-stained sections of the LV wall. Scale bars, 100 µm. Quantitative analysis of (**b**) myocyte cross-sectional area and (**d**) perivascular fibrosis and in the left ventricle of the six groups. Data are mean ± SEM of 7 rats per group. *P < 0.05 versus the WKY control group. ^†^*P* < 0.05 versus the SHR control group. Comparisons among three groups were tested using ANOVA followed by Dunnett’s multiple comparison tests using the JMP (version 8.0) software.

Light microscopic analysis demonstrated that the cross-sectional area of cardiomyocytes in the left ventricles was significantly greater in SHR than WKY (Fig. 2a,b). The cross-sectional area of cardiomyocytes was significantly greater in the CS high-dose rats than control rats in both strains (Fig. 2a,b). There were no significant differences in the cross-sectional area of cardiomyocytes after exposure to CS at low dose in both strains (Fig. 2a,b). The extent of perivascular fibrosis was significantly higher in high-dose SHR than the control WKY, but there were no significant differences between the other groups (Fig. 2c,d).

### Overall patterns of changes in protein expression

**Figure 3:**
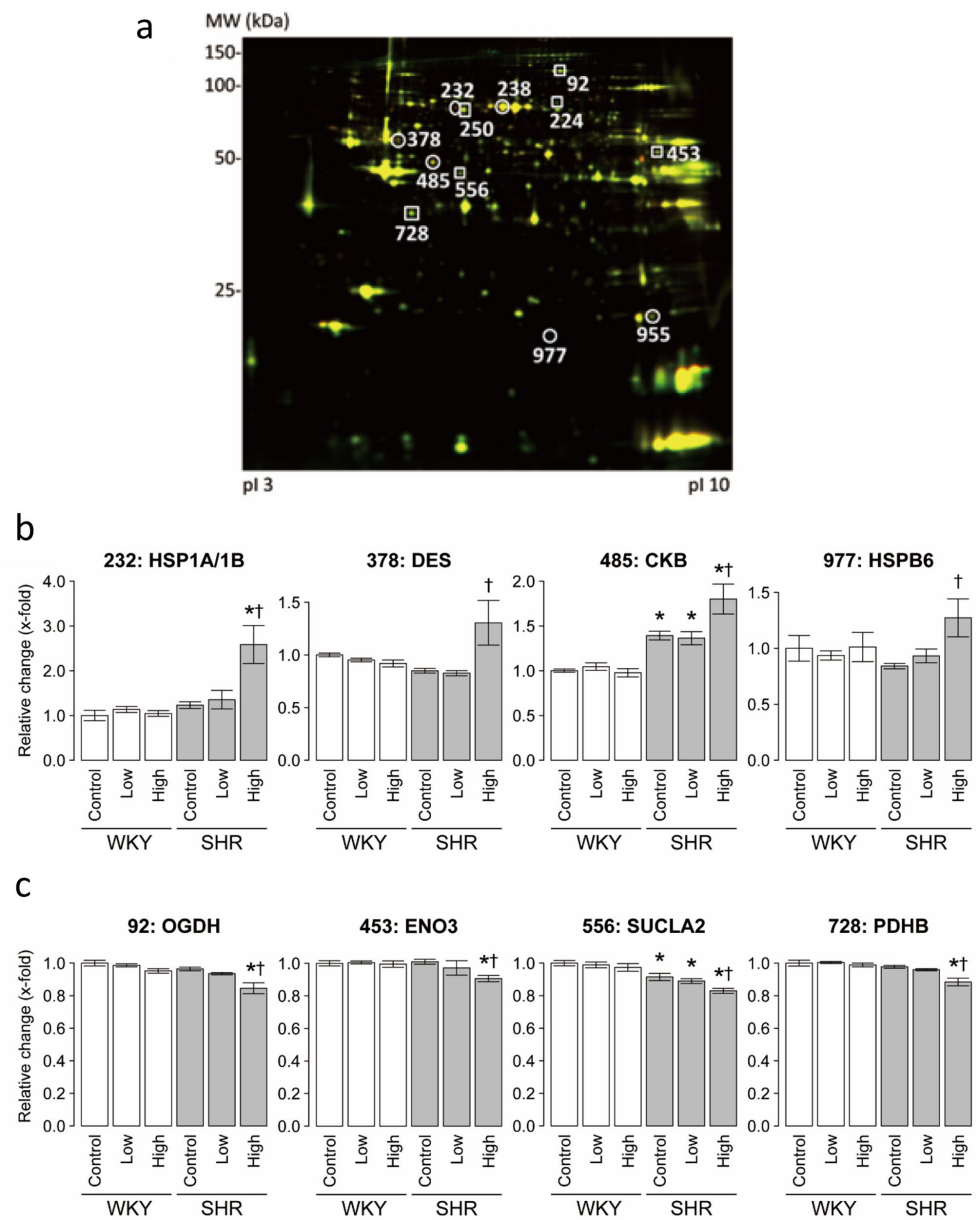
Representative 2D-DIGE image of LV lysates and expression levels of the identified proteins in the six groups. (**a**) Proteins (40 μg each) were labeled with Cy3 and Cy5 dyes, mixed and subjected to 2D-DIGE analysis. Cy3- and Cy5-images are illustrated using red and green pseudocolors, respectively. IPG strips (pI 3–11) were used for IEF, and 12.5% SDS-PAGE for the second dimension. The expression levels of LV proteins with significant (**b**) up-regulation and (**c**) down-regulation were quantified in the six groups. Data are mean ± SEM of 6 rats per group. *P < 0.05 versus the WKY control group. ^†^*P* < 0.05 versus the SHR control group. Numbers above each leaf represent the spot number for each protein. Comparisons among three groups were tested using ANOVA followed by Dunnett’s multiple comparison tests using the JMP (version 8.0) software.

The proteins were extracted and subjected to comparative analysis by 2D-DIGE. Figure 3a shows representative 2D-DIGE image of heart lysates of SHR control and CS high-dose SHR. Proteomic analysis by the DeCyder software analysis identified significant changes in 43 protein spots in the left ventricles of the latter group, compared with SHR control, with an absolute ratio of more than 1.1-folds with statistical significance (*p* < 0.05). Of the 43 spots in the 2-DE gels, 30 were isolated and subjected to MALDI-TOF/TOF MS. Figure 3b,c shows the relative changes in representative up-regulated and down-regulated 4 spots among the identified 30 spots in six groups. The changes in 30 protein spots in the heart were significantly different between the SHR control and CS high-dose SHR while the expression in 14 protein spots were significantly up-regulated in the CS high-dose SHR, compared to the SHR control. The 14 up-regulated spots in CS high-dose SHR were identified as heat shock 70 kDa protein 1A/1B (HSPA1A/1B; HSP70) (no. 232), serum albumin (ALB) (no. 238, 249, and 277), pyruvate kinase (PKM) (no. 335), desmin (DES) (no. 378 and 380), dihydrolipoyllysine-residue succinyltransferase component of 2-oxoglutarate dehydrogenase complex (DLST) (no. 385), creatine kinase B-type (CKB) (no. 485), acyl-coenzyme A thioesterase 2 (ACOT2) (no. 567), carbonic anhydrase 1 (CA1) (no. 851), alpha-crystallin B chain (CRYAB) (no. 955 and 957), and heat shock protein beta-6 (HSPB6; HSP20) (no. 977) (Table 2). Furthermore, the 16 protein spots with significant down-regulation in the CS high-dose SHR were identified as 2-oxoglutarate dehydrogenase (OGDH) (no. 92), mitochondrial inner membrane protein (IMMT) (no. 180), succinate dehydrogenase [ubiquinone] flavoprotein subunit (SDHA) (no. 224), dihydrolipoyllysine-residue acetyltransferase component of pyruvate dehydrogenase complex (DLAT) (no. 250), aldehyde dehydrogenase (ALDH2) (no. 433), beta-enolase (ENO3) (no. 453), succinyl-CoA ligase [ADP-forming] subunit beta (SUCLA2) (no. 550 and 556), long-chain specific acyl-CoA dehydrogenase (ACAD) (no. 596 and 605), NADH dehydrogenase [ubiquinone] 1 alpha subcomplex subunit 10 (NDUFA10) (no. 638), pyruvate dehydrogenase E1 component subunit beta (PDHB) (no. 728), myozenin-2 (MYOZ2) (no. 769), protein deglycase DJ-1 (PARK7) (no. 926), superoxide dismutase [Mn] (SOD2) (no. 944), and ATP synthase subunit d (ATP5H) (no. 951) (Table 2).

**Table 2:** List of proteins that showed significant differences in their myocardial expression levels in WKY and SHR.

Spot #	Symbol	Protein name	WKY		SHR	
			Fold change up (+)/down (−)		Fold change up (+)/down (−)	
			CTL-low	CTL-high	CTL-low	CTL-high
**Up-regulation**						
232	HSPA1A/1B	Heat shock 70 kDa protein 1A/1B	1.14	1.05	1.10	2.10
238	ALB	Serum albumin	− 1.01	− 1.04	− 1.01	1.25
249	ALB	Serum albumin	− 1.03	− 1.05	− 1.03	1.26
277	ALB	Serum albumin	1.07	− 1.06	− 1.07	1.31
335	PKM	Pyruvate kinase	1.07	1.00	1.04	1.12
378	DES	Desmin	− 1.05	− 1.08	− 1.03	1.54
380	DES	Desmin	− 1.02	− 1.06	− 1.03	1.52
385	DLST	Dihydrolipoyllysine-residue succinyltransferase component of 2-oxoglutarate dehydrogenase complex, mitochondrial	− 1.04	− 1.03	− 1.02	1.15
485	CKB	Creatine kinase B-type	1.05	− 1.02	− 1.02	1.29
567	ACOT2	Acyl-coenzyme A thioesterase 2, mitochondrial	− 1.02	1.04	1.02	1.06
851	CA1	Carbonic anhydrase 1	− 1.02	− 1.07	1.06	1.16
955	CRYAB	Alpha-crystallin B chain	1.04	1.01	1.00	1.11
957	CRYAB	Alpha-crystallin B chain	1.05	1.03	1.05	1.17
977	HSPB6	Heat shock protein beta-6	− 1.07	1.01	1.11	1.51
**Down-regulation**						
92	OGDH	2-oxoglutarate dehydrogenase, mitochondrial	− 1.01	− 1.05	− 1.03	− 1.14
180	IMMT	Mitochondrial inner membrane protein, mitochondrial	1.00	− 1.02	− 1.01	− 1.08
224	SDHA	Succinate dehydrogenase [ubiquinone] flavoprotein subunit, mitochondrial	1.01	− 1.07	1.01	− 1.17
250	DLAT	Dihydrolipoyllysine-residue acetyltransferase component of pyruvate dehydrogenase complex, mitochondrial	1.00	− 1.02	− 1.03	− 1.09
433	ALDH2	Aldehyde dehydrogenase, mitochondrial	− 1.03	− 1.11	− 1.05	− 1.11
453	ENO3	Beta-enolase	1.00	1.00	− 1.04	− 1.11
550	SUCLA2	Succinyl-CoA ligase [ADP-forming] subunit beta, mitochondrial	1.00	1.00	− 1.02	− 1.11
556	SUCLA2	Succinyl-CoA ligase [GDP-forming] subunit beta, mitochondrial	− 1.01	− 1.03	− 1.03	− 1.10
596	ACADL	Long-chain specific acyl-CoA dehydrogenase, mitochondrial	1.05	1.02	− 1.02	− 1.12
605	ACADL	Long-chain specific acyl-CoA dehydrogenase, mitochondrial	− 1.03	− 1.04	− 1.05	− 1.17
638	NDUFA10	NADH dehydrogenase [ubiquinone] 1 alpha subcomplex subunit 10, mitochondrial	− 1.10	− 1.05	− 1.01	− 1.07
728	PDHB	Pyruvate dehydrogenase E1 component subunit beta, mitochondrial	1.00	− 1.01	− 1.02	− 1.11
769	MYOZ2	Myozenin-2	1.04	1.05	− 1.04	− 1.09
926	PARK7	Protein deglycase DJ-1	1.03	− 1.01	− 1.01	− 1.04
944	SOD2	Superoxide dismutase [Mn], mitochondrial	− 1.01	1.00	1.03	− 1.07
951	ATP5H	ATP synthase subunit d, mitochondrial	1.00	1.00	1.00	− 1.08

*WKY *Wistar Kyoto rats, *SHR* Spontaneously Hypertensive rats, *CTL *control. The excised proteins were identified through protein database search by the Paragon Method using Protein Pilot software (Thermo Fisher Scientific).

### Functional categories of identified proteins

**Figure 4:**
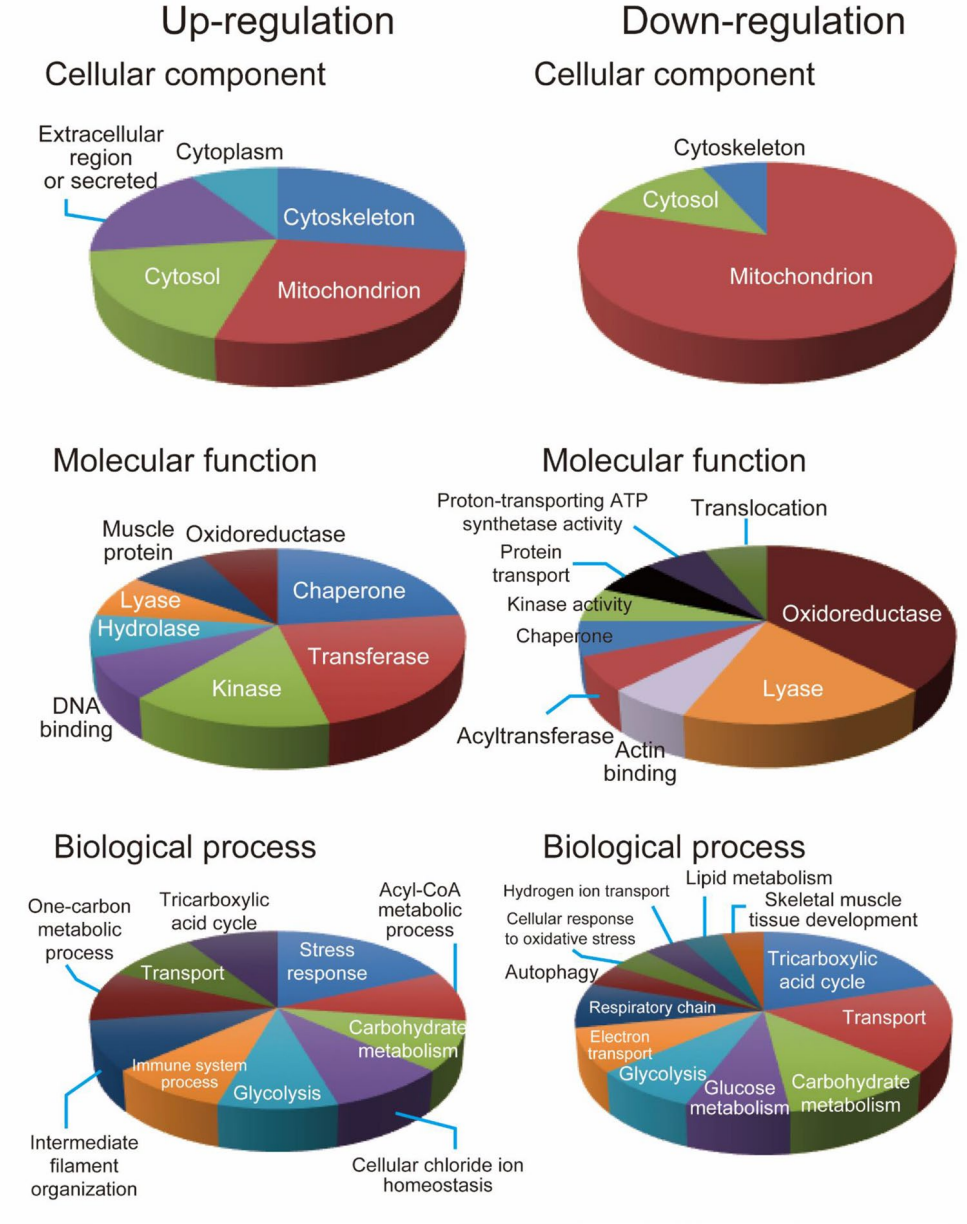
Protein ontology classification of identified proteins. The graphs show the percentages of corresponding protein ontology terms to the total number of annotated proteins. The identified up-regulated or down-regulated proteins were grouped according to their associated cellular components, molecular functions, and biological processes.

To understand the functional roles of the 30 proteins in the CS high-dose SHR they were assigned to UniProt database. Figure 4 shows the proportion of groups assigned according to their associated biological processes and molecular functions in each of the up-regulated or down-regulated proteins. Mapping for the cellular components showed that the down-regulated proteins were mainly mitochondrial (Fig. 4). The molecular functions of these proteins were mainly chaperone and oxidoreductase activities. Furthermore, annotation for the biological processes showed that the altered proteins belonged to stress response or tricarboxylic acid cycle (Fig. 4).

### Expression of identified proteins

**Figure 5:**
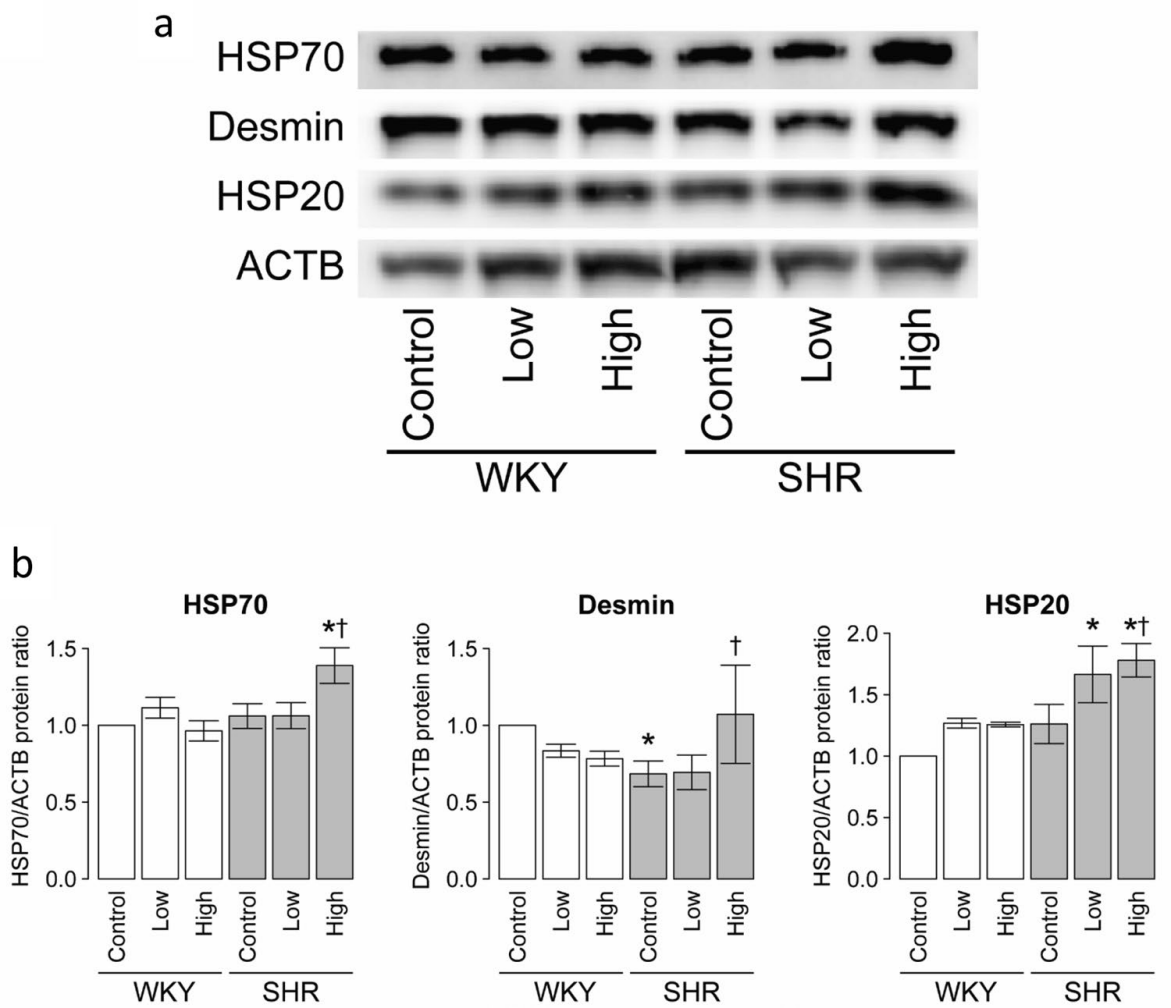
Amounts of HSP70, desmin, and HSP20 in six groups. (**a**) Representative immunoblots of each protein. (**b**) The amount of each protein quantitated relative to the amount of β-actin (ACTB) and expressed relative to the value of WKY controls. Data are mean ± SEM of 6 rats per group. *P < 0.05 versus the WKY control group. ^†^*P* < 0.05 versus the SHR control group. Comparisons among three groups were tested using ANOVA followed by Dunnett’s multiple comparison tests using the JMP (version 8.0) software.

As the expression of protein no. 232, 378, 380, and 977 was significantly higher in CS high-dose SHR and the control SHR, we performed next western blot analysis on HSP70 (no. 232), desmin (no. 378 and 380), and HSP20 (no. 977) to confirm the results of proteomics analysis. The expression of HSP70, desmin, and HSP20 was significantly up-regulated in SHR exposed to high-dose CS, compared with the control SHR (Fig. 5). These findings were consistent with those of proteomic analysis.

## Discussion

In the present study, we performed proteomic analysis to identify the molecules associated with CS-induced cardiac hypertrophy in rats. The results showed that high-dose CS for 2 months significantly changed the amount of 43 proteins in the left ventricles. Among these proteins, proteomic analysis identified 30 protein spots that showed significant alterations, with 14 up-regulated and 16 down-regulated proteins in the left ventricles of SHR subjected to long-term high-dose CS, compared with control SHR. Among the altered proteins, HSP70 and HSP20 showed significant up-regulation in the left ventricles.

Our results showed that progression of cardiac hypertrophy in CS-exposed SHR was more prominent than in their WKY counterparts. Cardiac hypertrophy was assessed by the increases in LV weight and cross-sectional area of left ventricles after exposure to CS. Previous experimental studies have demonstrated that cigarette smoking causes endothelial dysfunction and cardiac hypertrophy through cellular oxidative stress and decreased NO bioavailability^25^. Cigarette smoking is also known to induce mitochondrial oxidative stress, which contributes to the development of hypertension^17^. On the other hand, one previous study reported that CS can induce cardiac hypertrophy without any increase in blood pressure^14^. Consistently with the finding of that study, our results showed that exposure of SHR to CS induced cardiac hypertrophy but did not affect blood pressure, suggesting that while CS may induce pressure overload consequent to its effect on blood pressure, it has direct effect on the myocardium.

Two members of the heat shock proteins (HSP70 and HSP20) showed significant up-regulation in the left ventricles of CS high-dose SHR. The previous study has also shown that exposure to CS produces cellular injury as well as cytoprotective stress proteins, including heat shock proteins^26^. Heat shock protein (HSP) family is a molecular chaperone known to be up-regulated in cellular stress/damage. The increase in HSP expression observed in the present study could serve to protect against the effects of excessive stress on the myocardium caused by CS. Among the members of HSP, HSP70 1A/1B is a high molecular weight HSP family known to participates in the protection of the myocardium during cardiovascular stress by preventing or reversing abnormal protein folding or aggregation^27^. Kee, et al.^28^ reported that stress signals, such as hypertrophic stimuli and reactive oxygen species (ROS) to cardiomyocytes induce the expression of HSP70, followed by activation of cardiac histone deacetylase-2 (HDAC2) that triggers cardiac hypertrophy due to inhibition of antihypertrophic gene expression. In addition, Yoon, et al.^29^ have shown that inhibition of HSP70 blocked the development of cardiac hypertrophy. These findings highlight the pathological importance of HSP70 in cardiac hypertrophy. HSP20 belongs to a small molecular weight HSP family, and is widely recognized as the main mediator of cardioprotective signaling^30^. HSP20 is highly expressed in different types of muscles, including vascular, cardiac, skeletal and smooth muscles^31^ and is functionally known to protect against cardiac ischemia–reperfusion injury, myocardial hypertrophy, and β-agonist-induced cardiomyocyte apoptosis^31^. HSP20 forms a complex with protein kinase D1 (PKD1) and regulates the hypertrophic signaling pathway involved in the induction of fetal gene program, pathological cardiac growth, and cardiac remodeling^32^. It is also reported that cardiac-specific overexpression of HSP20 in a transgenic mouse model enhanced contractility and improved myocardial function^33^. Thus, enhanced HSP20 might protect cardiac function against CS-induced cardiac hypertrophy by modulating cardiac contractility and apoptosis. These reports supported our suggestion that higher HSP70 and HSP20 levels play an important role in regulation of the CS-induced cardiac hypertrophy.

Desmin is a cytoskeletal intermediate filament protein and an integral component of the cardiomyocyte^34^. The desmin network, which forms a filamentous lattice in the interfibrillar space to anchor myofibers at the Z-discs and maintains their alignment within myocytes, serves to maintain the structural and functional integrity of myocytes^34^. While deficiency of desmin is associated with cardiomyopathy and myocyte destruction, high levels of desmin or disorganization of desmin filaments have been found in cardiac hypertrophy and heart failure^35–37^. It is possible that aberrant aggregation of desmin and disruption of the desmin network enhance contractility by remodeling the heart, and further worsen cardiac function^38^. In addition, desmin accumulation correlates with the remodeling process and cardiac performance^39^. Although there has been no report of CS-induced up-regulation of desmin expression in the myocardium, the increased desmin might play an important role in CS-induced cardiac hypertrophy.

Cigarette smoking induces excessive generation of ROS^14^. In the present study, the UniProt analysis and mapping of protein expression reveal that several components of mitochondria were up-regulated and down-regulated in CS high-dose SHR (Fig. 4). Furthermore, many of these proteins were oxidoreductases and lyases known to be involved in the tricarboxylic acid cycle. Cigarette smoking induces oxidative damage in cardiovascular mitochondria, and disturbs the functions of the mitochondrial respiratory chain and cellular ATP production, leading to myocardial contractile dysfunction and hypertension^19,40^. In addition, it has been reported that CS-exposed rats showed significantly low activities of tricarboxylic acid cycle enzymes and mitochondrial enzymatic antioxidants and increased lipid peroxidation in the mitochondria^41^. The toxicity of CS may target the mitochondria of cardiomyocytes, and causes cardiac dysfunction by changing the expression level of mitochondrial proteins.

In the present study, body weight was significantly bigger in WKY than in SHR before exposure to CS, but there was no difference in body weight between the three groups in each of WKY and SHR. After exposure to CS, LV weight was significantly increased in SHR. These results indicated that cardiac hypertrophy was caused by exposure to CS in SHR. Even in WKY, LV weight was increased by exposure to CS, but this increase did not reach the level of statistical significance at the end of the 2-month exposure. Exposure to CS for longer period might induce obvious cardiac hypertrophy in WKY. In addition, the cross-sectional area of cardiomyocytes was significantly increased after exposure to CS in both strains. However, the extent of perivascular fibrosis was increased, but there were no significant differences between controls and CS-exposed rats statistically. CS might affect myocytes rather that cardiac fibroblasts. It has been reported that SHR is susceptible to oxidative stress in several organs compared with WKY^40,42^. Cigarette smoking is also known to induce mitochondrial oxidative stress^17^. Therefore, it is considered that SHR are more responsive to exposure to CS not only in the heart but also in other organs, including the kidneys and lungs, compared with WKY. Further studies are needed to clarify the mechanisms of CS-induced cardiac hypertrophy and the susceptibility to CS in the organs of SHR compared with WKY.

## Conclusion

Cigarette smoking for 2 months induced cardiac hypertrophy without any increase in blood pressure in SHR. Proteomics analysis identified significant up-regulation of HSP70 and HSP20 in the left ventricles of these rats after exposure to CS. One of limitations of the present study is that the evaluation of cardiac function using echocardiographic or cardiac magnetic resonance studies was not performed although we observed cardiac hypertrophy induced by cigarette smoking. Nevertheless, our findings suggest that HSP might be the main mediators of CS-induced cardiac hypertrophy. As further study, loss of function studies for HSP70 and HSP20 are needed to determine the potential role of these proteins in CS-induced cardiac hypertrophy.

## Methods

### Animal experiments

Male Wistar Kyoto (WKY) and spontaneously hypertensive rats (SHR) were provided from the Disease Model Cooperative Research Association, Kyoto Japan^43^ and used in this study at 10 weeks of age. Rats were exposed to CS at low (2 puffs/min for 40 min/day) or high dose (2 puffs/min for 120 min/day) (n = 7 per group) for 5 days a week for 2 months. The CS inhalation was conducted using unfiltered research cigarette 3R4F (Tabacco Health Research Institute, Kentucky University, Lexington, KY) and the Tabacco Smoke Inhalation Experiment System for small animals (Model SIS-CS; Shibata Scientific Technology, Tokyo, Japan)^44^. The smoke generator automatically generated CS by setting the volume of the syringe pump and the number of puffs per minutes. The generated CS was delivered to the inhalation chamber to which the rat body holders were set and rats were exposed to CS through the nose. The CS protocol mimicked the modified Federal Trade Commission protocol at a rate of 2 puffs/min, 2 s/puff, and 35 mL/puff^45^. The rats were fed normal diet and housed in a temperature-controlled (25ºC) environment with a 12-h light–dark cycle. Body weight was measured each week. The experimental protocol was approved by the animal care committee of Mie University and all animal procedures were conducted humanely in accordance with the guidelines for the care and use of laboratory animals approved by the university. The investigation conformed to the Guide for the Care and Use of Laboratory Animals published by the US National Institutes of Health (NIH Publication No. 85-23, revised 1996).

### Measurement of blood pressure

Systolic blood pressure was measured in conscious rats by the tail-cuff method using BP-98A, MCP-1 (Softron, Tokyo) at the end of CS exposure using the method described previously^46^. The reported blood pressure and heart rate values represent the mean of four or five determinations taken at the same time period. After measurement of the blood pressure, the rats were sacrificed by deep pentobarbital anesthesia.

### Tissue sample collection and histological analysis

The heart was carefully dissected out and weighed. The left ventricle was separated from atria and right ventricles, weighed, and cut into 2-mm-thick slices. The apex and base of the left ventricles were then immediately frozen in liquid nitrogen for quantitative real-time polymerase chain reaction (RT-PCR) analysis and proteomics analysis. Midventricular slices were fixed in 4% paraformaldehyde in phosphate-buffered saline (PBS). The heart tissues were embedded in paraffin, sectioned (thickness, 4 µm), and stained with hematoxylin eosin solution to measure the cross-sectional area of myocardium and with Sirius red solution to evaluate the extent of fibrosis, as described previously^47^. The cross-sectional areas of myocardium and the area of the fibrosis in the perivascular region were calculated with the cellSens image system (Olympus, Tokyo, Japan).

### Quantitative assessment of cardiac hypertrophy

Total RNA was extracted from LV tissue (n = 7 per group) using the ReliaPrep RNA Tissue Miniprep System (Promega, Madison, WI), according to the instructions provided by the manufacturer. The concentration of total RNA was quantified by spectrophotometry (ND-1000; NanoDrop Technologies, Wilmington, DE). RNA was reverse transcribed to single-strand cDNA using SuperScript III First-Strand Synthesis System for RT-PCR (Thermo Fisher Scientific, Waltham, MA). The cDNA was subjected to quantitative PCR analysis with FastStart Universal Probe Master Mix (Roche, Basel, Switzerland) using primers specific for mRNAs encoding atrial natriuretic peptide (ANP), brain natriuretic peptide (BNP), collagen I, and collagen III genes, with an ABI 7300 Real-Time PCR system (Thermo Fisher Scientific), as described previously^48^. Standard curves were generated for each primer set. The gene expression level was normalized to that of β-actin (ACTB) in the same cDNA. All experiments were performed in duplicates.

### Preparation of heart protein samples

Frozen LV tissues were homogenized in lysis buffer (30 mM Tris–HCl, 7 M urea, 2 M thiourea, 4% w/v CHAPS, and a cocktail of protease inhibitors, pH 8.5). After incubation for 60 min on ice, homogenates were centrifuged at 30,000 × *g* for 30 min at 4 °C and the supernatant was collected. The protein concentration in the supernatant was determined by the BCA protein Kit (Thermo Fisher Scientific), using bovine serum albumin as a standard.

### Two-dimensional fluorescence differential gel electrophoresis (2D-DIGE)

Each sample was labeled with amine-reactive cyanine dyes, Cy3 or Cy5 developed for fluorescence 2D-DIGE technology (GE Healthcare, Little Chalfont, UK). The heart tissue mixture was labeled with Cy2 to be used as an internal standard and 2-DE was performed as described previously^49^. After 2-DE, cyanin-labeled proteins were visualized directly by scanning, using a Typhoon 9400 imager (GE Healthcare) in fluorescence mode.

### Protein identification

Protein spots with *p* < 0.05 in all three comparisons were selected for further identification. The selected spots were excised manually from Coomassie Brilliant Blue (CBB) R-350 (PhastGel Blue R, GE Healthcare)-stained preparative 2-DE gels using a gel spot cutter. Then, the protein samples were in-gel digested using the protocol described previously^50^. The peptide mixtures were analyzed by a matrix-assisted laser desorption ionization time-of-flight tandem mass spectrometry (MALDI-TOF/TOF MS; 4800 *Plus* MALDI TOF/TOF™ Analyzer; Thermo Fisher Scientific) operating in positive-ion reflector mode. The excised proteins were identified through protein database search by the Paragon Method using Protein Pilot software (Thermo Fisher Scientific).

### Western blot analysis

Western blot analysis was conducted to confirm the results of proteomic analysis. The LV tissues collected after exposure to CS were homogenized in lysis buffer (T-PER reagent; Thermo Fisher Scientific). Samples (n = 6 per group) were separated by 12% SDS-PAGE and electroblotted onto polyvinylidenedifluoride (PVDF) membranes (Merck Millipore, Burlington, MA). The membranes were incubated with mouse monoclonal antibody to heat shock protein (HSP) 70 (Abcam, Cambridge, UK) at 1:1000 dilution and rabbit monoclonal antibodies to desmin (Abcam) at 1:100,000 dilution and HSP20 (Abcam) at 1:10,000 dilution. Mouse anti-β-actin (ACTB) monoclonal antibody (Merck Millipore) at 1:5000 dilution was used as the loading control. The protein bands were visualized by ECL plus Western blotting detection system (GE Healthcare) and Quantity One v3.0 software (Bio-Rad Laboratories, Hercules, CA) was used to quantitate the band intensities. Protein expression levels were normalized relative to the level of β-actin protein in the same tissue sample (Supplementary information).

### UniProt analysis and mapping of protein expression

Protein ontology classification was performed by importing proteins into the protein analysis using the Universal Protein Resource (UniProt) databases (https://www.uniprot.org/; European Bioinformatics Institute, Cambridge, UK: SIM Swiss Institute Bioinformatics, Geneva, Switzerland: Protein Information Resource, Washington, DC, USA). Proteins were grouped according to their associated biological processes and molecular functions.

### Statistical analysis

Data are presented as mean ± standard error of the mean (SEM). Comparisons among three groups were tested using one-way analysis of variance (ANOVA) followed by Dunnett’s multiple comparison tests using the JMP 8.0 software (SAS Institute Inc, Cary, NC). A *P* value < 0.05 was considered statistically significant.

## Supplementary information

Supplementary Information.
