# Supplementary material for: Proteomic identification of the proteins related to cigarette smoke-induced cardiac hypertrophy in spontaneously hypertensive rats
Source: Sci Rep. 2020 Nov 2;10:18825. doi: 10.1038/s41598-020-75429-3 (PMC7608641; doi:10.1038/s41598-020-75429-3)
Supplement: Supplementary file 1 — Supplementary Information. [file 41598_2020_75429_MOESM1_ESM.docx]

**Proteomic identification of the proteins related to cigarette smoke-induced cardiac hypertrophy in spontaneously hypertensive rats**

Yuki Kitamura^1,†^, Nathan Mise^2^, Yurie Mori^1^, Yuka Suzuki^3^, Tomoki Ohashi^3^, Saeko Tada-Oikawa^3,‡^, Masaki Tokisu^4^, Cai Zong^4^, Shinji Oikawa^1^, Sahoko Ichihara^2,3,*^

^1^Department of Environmental and Molecular Medicine, Mie University Graduate School of Medicine, Tsu, Japan; ^2^Department of Environmental and Preventive Medicine, Jichi Medical University School of Medicine, Shimotsuke, Japan; ^3^Graduate School of Regional Innovation Studies, Mie University, Tsu, Japan; ^4^Department of Occupational and Environmental Health, Tokyo University of Science, Noda, Japan


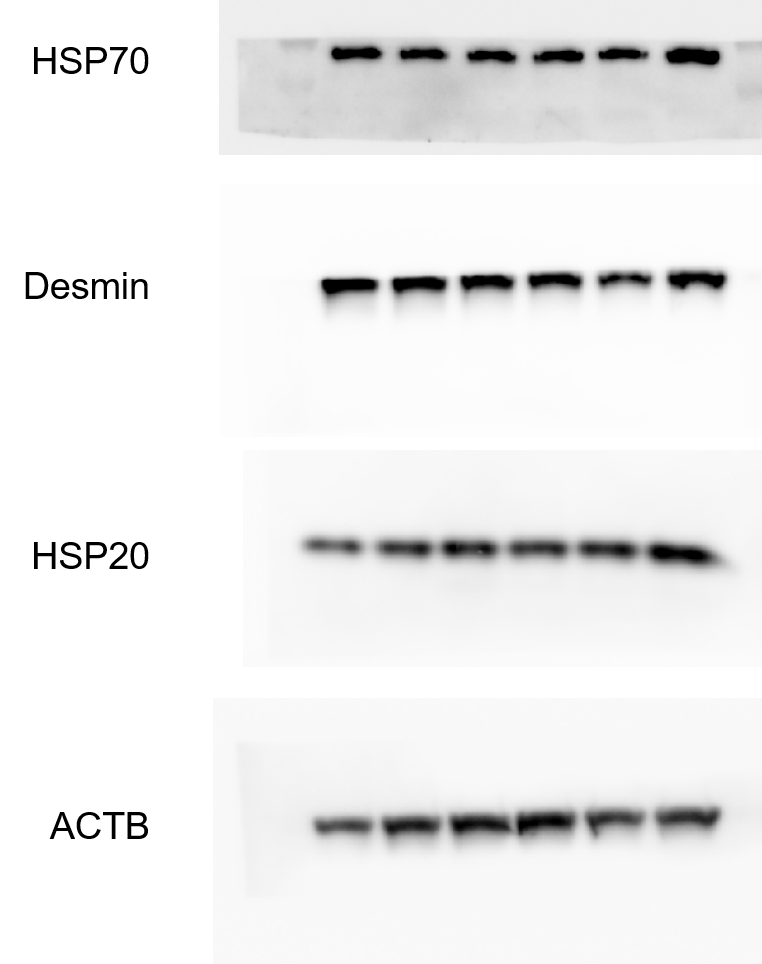


**Suppl. Figure 1.** The blot images on full length membrane used in Figure 5.
